# Differential Host Responses and Viral Replication of Highly Pathogenic Avian Influenza H5N1 Strains in Diverse Cell Lines with a Raw Milk Supplement

**DOI:** 10.3390/life15101625

**Published:** 2025-10-17

**Authors:** Gagandeep Singh, Patricia Assato, Isaac Fitz, Sujan Kafle, Juergen A. Richt

**Affiliations:** Department of Diagnostic Medicine/Pathobiology (DMP), College of Veterinary Medicine, Kansas State University, Manhattan, KS 66506, USA

**Keywords:** bovine-H5N1, raw milk, host transcriptomics, mink-H5N1

## Abstract

The highly pathogenic avian influenza (HPAI) H5N1 virus poses a growing global health threat, particularly following its unprecedented spillover into dairy cattle and subsequent transmission to more than 1000 dairy farms in 18 states. This study investigates the host cell responses to distinct H5N1 strains (bovine- and mink-derived H5N1) in the presence and absence of raw milk across diverse mammalian cell lines (MDCK, MDBK, A549, Vero, MV1). Our findings reveal that the bovine-derived H5N1 strain exhibits more robust replication than the mink-derived H5N1 and demonstrates intra-host viral evolution with emerging amino acid substitutions detectable by deep sequencing. Although raw milk supplementation did not directly enhance viral replication in vitro, it significantly modulated host gene expression, often dampening key antiviral interferon-stimulated gene (ISG) responses and disrupting essential host cellular processes like intracellular trafficking and sialic acid biosynthesis. These host gene modulations are cell-type- and strain-specific, suggesting a complex interplay that may theoretically influence virus–host dynamics, though the biological significance of these in vitro observations requires validation through infectious virus assays and in vivo studies. This hypothesis-generating work provides preliminary insights into H5N1-milk interactions, highlighting the need for further mechanistic investigation to assess potential implications for viral transmission in dairy environments.

## 1. Introduction

Influenza A viruses (IAVs) represent a persistent and formidable challenge to global public health, primarily due to their remarkable capacity for rapid evolution [1,2,3]. This inherent genetic instability is fundamentally driven by the virus’s high mutation rate, which has been reported to be approximately 10^−5^ to 10^−6^ mutations per nucleotide per replication cycle (genetic drift), and by the ability to reassort (genetic shift) [4,5,6]. Within the diverse landscape of influenza A viruses, highly pathogenic avian influenza (HPAI) H5N1 viruses of the goose/Guangdong lineage have emerged as a particularly serious concern [7]. First identified in China in 1996, this lineage rapidly disseminated across the globe, causing devastating outbreaks with acute mortality in domestic poultry and wild bird populations [7,8]. Since 2021, the H5N1 clade 2.3.4.4b has spread globally, leading to sporadic infections and fatal outbreaks in diverse mammalian species, including sea lions, cats, and mink, further underscoring its expanding host range [9,10,11,12,13,14,15,16]. While many of these infections are considered “dead-end” in terms of onward mammalian transmission, they nonetheless provide opportunities for viral adaptation to a mammalian host [17]. However, the zoonotic threat posed by H5N1 viruses took an unprecedented turn with its recent spillover into dairy cattle and the maintenance of transmission from cow to cow [18,19,20]. The strong tropism for bovine mammary glands and the resulting high viral loads in milk represent a critical change in the virus’s behavior [18,19,20]. The presence of specific sialic acid receptors for both avian and mammal IAVs in bovine mammary gland tissue provides the basis for this unique tropism, enabling efficient viral replication within the cow udder and transmission via milking-associated procedures [21,22,23]. This new transmission pathway creates novel and augmented public health risks. It directly exposes dairy farm workers to H5N1 and raises significant food safety concerns regarding the consumption of raw milk. Moreover, the documented infection of other mammals (e.g., cats, mice) through raw milk consumption underscores the potential for dairy farms to act as vehicles for further spillover events, exacerbating the overall zoonotic threat of H5N1 [24,25].

Despite the escalating global threat posed by HPAI H5N1 and the unprecedented nature of its spillover into dairy cattle, a detailed understanding of the molecular mechanisms by which host cells respond to different H5N1 strains in the unique biological context of milk remains incomplete. Furthermore, comparative analyses of host responses to distinct H5N1 clade 2.3.4.4b strains, such as those recently isolated from bovine and mink hosts, are crucial. Such comparisons can identify strain-specific uniqueness and their differential impacts on cellular processes, which are vital for informing accurate risk assessments and developing targeted intervention strategies. To address this critical knowledge gap, we hypothesize that the presence of raw milk components in in vitro mammalian cell cultures will alter host–cell antiviral responses and potentially affect H5N1 virus replication patterns compared to standard cell culture conditions. Specifically, we predict that (1) H5N1-infected cells exposed to raw milk will exhibit modified interferon-stimulated gene (ISG) expression profiles compared to controls, (2) viral replication kinetics may differ between milk-treated and untreated conditions, and (3) these effects may vary between different mammalian cell lines representing various host species. This hypothesis-generating study aims to explore these potential interactions to inform future mechanistic and in vivo investigations of H5N1 transmission dynamics in dairy environments.

## 2. Materials and Methods

### 2.1. Cells and Viruses

Madin-Darby Canine Kidney (MDCK, ATCC, cat# CCL-34), Madin-Darby Bovine Kidney (MDBK, ATCC, cat# CCL-22), A549 (human lung carcinoma, ATCC, cat# CCL-185), MV1 (mink lung epithelial, ATCC, cat# CCL-64), and Vero (African green monkey kidney, ATCC, cat# CCL-81) cells were obtained from ATCC. All cell lines were cultured in Dulbecco’s Modified Eagle Medium (DMEM) (ThermoFisher Scientific, Waltham, MA, USA), supplemented with 10% fetal bovine serum (FBS) (ThermoFisher Scientific, Waltham, MA, USA), and 1X antibiotic-antimycotic solution (ThermoFisher Scientific, Waltham, MA, USA) containing 100 U/mL penicillin, 100 µg/mL streptomycin, and 0.25 µg/mL amphotericin B. Cells were maintained at 37 °C in a humidified atmosphere with 5% CO_2_.

The highly pathogenic avian influenza virus (HPAIV) A/dairy cattle/Kansas/5/2024 (bovine-H5N1) was isolated from an HPAIV-impacted dairy farm in Kansas. This virus was propagated in 9-day-old specific-pathogen-free (SPF) embryonated chicken eggs as previously described [26]. The HPAIV A/Mink/Spain/3691-8_22VIR10586-10/2022 (mink-H5N1) was kindly provided by Dr. Francesco Bonfante and Dr. Isabella Monne from the Istituto Zooprofilattico Sperimentale delle Venezie, Legnaro, Italy, and Dr. Monserrat Agüero and Dr. Azucena Sánchez from the Laboratorio Central Veterinario (LCV), Ministry of Agriculture, Fisheries and Food, Madrid, Spain, via Dr. Richard Webby from St. Jude Children’s Research Hospital, Memphis, TN. This mink-H5N1 strain originated from a significant outbreak in farmed mink in Spain in October 2022 [13]. All viral stocks were titrated by standard plaque assay on MDCK cells and stored at −80 °C until use [26].

### 2.2. Raw Milk Processing and Infection Medium Preparation

Fresh raw milk was collected from a bulk tank at the Dairy Teaching and Research Center, Department of Animal Sciences & Industry, Kansas State University, Manhattan, KS, USA. The raw milk was processed to remove somatic cells and most of the fat content by centrifugation at 400× *g* for 10 min at 4 °C as described previously [27]. Following centrifugation, the pelleted cell material and the top fat layer were carefully discarded. The remaining clarified raw milk was then sterile-filtered by passing it through a 0.8 µm Millex^®^ MCE syringe filter (Millipore Sigma, Burlington, MA, USA).

Two types of infection media were prepared for this experiment:

1. Normal Infection Medium: Prepared by mixing sterile cell culture grade water (ThermoFisher Scientific, Waltham, MA, USA) 1:1 with 2X Minimum Essential Medium (MEM) (Millipore Sigma) containing 2X antibiotic-antimycotic solution (ThermoFisher Scientific, Waltham, MA, USA).
2. Milk-Supplemented Infection Medium: Prepared by mixing the processed raw milk (as described above) 1:1 with 2X MEM (Millipore Sigma, Burlington, MA, USA) containing 2X antibiotic-antimycotic solution (ThermoFisher Scientific, Waltham, MA, USA).

### 2.3. Virus Infections

Cells were seeded at a density of 1 × 10^6^ cells/well in 6-well plates (Corning, Corning, NY, USA) and incubated for 4 h at 37 °C in 5% CO_2_ to allow for cell adherence and minimize differences in cell numbers due to varying cell line growth kinetics. Following the 4 h incubation, the cell culture medium was aspirated, and cells were infected at a multiplicity of infection (MOI) of 0.01 with either bovine-H5N1 or mink-H5N1. Each virus was diluted in either the normal infection medium or the milk-supplemented infection medium, as prepared above. A volume of 1 mL of the virus inoculum was added per well. Following virus adsorption, cells and viruses were incubated together for 24 h at 37 °C in 5% CO_2_ for subsequent experiments. For uninfected controls, cells were incubated with the respective infection media without virus.

### 2.4. RNA Extraction

Total RNA was extracted from infected and uninfected cells using an automated bead extraction method with modifications from a previously described protocol [26]. Briefly, after the 24 h infection period, the infection media were removed from the cells, and the cell monolayers were washed three times with 1X phosphate-buffered saline (PBS) (ThermoFisher Scientific, Waltham, MA, USA). Adherent cells were then lysed by adding 150 µL of RLT lysis buffer (Qiagen, Hilden, Germany) and pipetting 10–12 times to ensure complete lysis. The cell lysate was incubated at room temperature for 10 min, followed by the addition of 150 µL of 1X PBS (ThermoFisher Scientific, Waltham, MA, USA).

For automated RNA extraction, 200 µL of the RLT lysate was utilized with the taco™ mini Automatic Nucleic Acid Extraction System (GeneReach, Taichung City 407, Taiwan) employing the Total NA extraction kit (Gene Reach, Taipei, Taiwan) according to the manufacturer’s instructions, with two specific modifications. Firstly, magnetic beads were introduced into the lysis buffer before the addition of the sample, followed by the addition of 200 µL of molecular-grade isopropanol (ThermoFisher Scientific, Waltham, MA, USA). Secondly, the final wash buffer B was substituted with 200-proof molecular-grade ethanol (ThermoFisher Scientific, Waltham, MA, USA). Each extraction yielded 100 µL of RNA in elution buffer. For cell culture supernatant analysis, 300 µL of the infection media were also processed using the same automated bead extraction method.

### 2.5. Reverse Transcription Real-Time PCR (RT-qPCR) Assays for Quantifying Influenza a Genome

RNA extracted from cells and supernatant was subjected to one-step RT-qPCR targeting the Influenza A Matrix gene, as described previously [26].

### 2.6. Determination of Virus Infectious Particles from Ct Values

To establish a quantitative relationship between RT-qPCR Ct values and viral infectivity, a standard curve was generated using the bovine H5N1 virus stock. The virus suspension was serially diluted ten-fold in infection medium (without milk supplementation) to create a dilution series spanning 8 orders of magnitude (neat to 1:10^8^). RNA was extracted from each dilution using the automated RNA extraction system described previously. The influenza A virus Matrix gene was quantified by one-step RT-qPCR performed in duplicate for each dilution, following the methodology outlined above. A standard curve correlating Ct values to known viral titers (log_10_ TCID_50_/mL) was constructed using linear regression analysis in Microsoft Excel (version 16.100.3). The standard curve demonstrated excellent linearity (R^2^ = 0.9779), and this relationship was used to convert all experimental Ct values to viral load equivalents expressed as log_10_ TCID_50_/mL. Complete standard curve data, including the regression equation, statistical parameters, and dilution series results, are presented in Supplementary Data.

### 2.7. RNA-Seq Library Preparation and Sequencing Using Illumina Short Read Technology

One microgram of each extracted total RNA sample was treated with DNase I (ThermoFisher Scientific, Waltham, MA, USA) according to the manufacturer’s instructions to remove any residual genomic DNA. After DNase treatment, RNA was quantified using the Qubit™ RNA Broad Range (BR) kit (ThermoFisher Scientific, Waltham, MA, USA) and a Qubit Fluorometer (ThermoFisher Scientific, Waltham, MA, USA). RNA integrity was evaluated using the TapeStation 4150 system (Agilent Technologies, Santa Clara, CA, USA) with RNA ScreenTape Analysis software version 5.1 (Agilent Technologies, Santa Clara, CA, USA), ensuring an RNA Integrity Number (RIN) value of >7.0 for all samples.

Samples were then normalized to a concentration of 10 ng/µL, and a total of 100 ng of RNA from each sample was used for library preparation. Libraries were prepared using the Illumina Stranded Total RNA Prep with Ribo-Zero Plus kit (Illumina, San Diego, CA, USA) following the manufacturer’s instructions. This kit depletes ribosomal RNA, allowing for the sequencing of mRNA and non-coding RNA. The prepared libraries were quantified again using the Qubit™ DNA Broad Range (BR) kit (ThermoFisher Scientific, Waltham, MA, USA), and the library sizes were determined using High Sensitivity D5000 ScreenTape (Agilent Technologies, Santa Clara, CA, USA) on the TapeStation 4150. The libraries were normalized to 4 nM and pooled. Paired-end sequencing was performed on an Illumina NextSeq platform (Illumina, San Diego, CA, USA) using the NextSeq 500/550 High Output Kit v2.5 with 150 Cycles reagent kit (Illumina, San Diego, CA, USA), generating 2 × 75 bp reads.

### 2.8. Bioinformatics Analysis

The sequencing process yielded raw FASTQ files, which were used for two distinct downstream analyses: Differential gene expression (DGE) and variant calling.

#### 2.8.1. Variant Calling Analysis

Total Raw sequencing reads were processed for variant calling using SeqMan Ultra Version 17.6 (DNASTAR, Madison, WI, USA).

1. Alignment: Samples from bovine-H5N1 infected cells were aligned against the full genome sequence of A/dairy cattle/Kansas/5/2024 (NCBI GenBank ID PP732373-80). Samples from mink H5N1-infected cells were aligned against the full genome sequence of A/Mink/Spain/3691-8_22VIR10586-10/2022 (GISAID ID EPI2220590-97) (Table 1).
2. Variant Detection: Variant detection parameters within SeqMan Ultra included a Minimum Score at SNP of 5.
3. Visualization: The results of the variant calling analysis were visualized using GraphPad Prism (version 10, GraphPad Software, Boston, MA, USA).

#### 2.8.2. Differential Gene Expression (DGE) Analysis

Raw sequencing reads were processed using the Galaxy platform hosted on the Kansas State University research computing cluster (BEOCAT).

1. Quality Control and Trimming: Raw sequences were first trimmed to remove adapter sequences and low-quality bases using Trimmomatic version 0.39 [28].
2. Alignment: Trimmed reads were aligned to species-specific reference genomes (Table 1) using HISAT2 version 2.1.0 [29].
3. Gene Counting: Aligned reads were then used to quantify gene expression by counting reads per transcript using StringTie version 2.2.1 [30].
4. Differential Expression Analysis: Raw gene counts were imported into R statistical software (R Core Team, 2024) and analyzed for differential gene expression using the DESeq2 package version 1.49.3 [31]. DESeq2 employs a negative binomial generalized linear model to identify genes with statistically significant changes in expression between experimental conditions. Genes with an adjusted *p*-value (FDR) < 0.05 and an absolute log_2_ fold change > 1 were considered differentially expressed.
5. Visualization: Boxplots showing the expression distributions of selected differentially expressed genes were generated in Python version 3.12.5 using the seaborn library [32].
6. Functional Enrichment Analysis: The top upregulated and downregulated genes were further analyzed for gene function enrichment using the Metascape online platform (https://metascape.org, accessed on 21 August 2024) [33]. Enriched pathways and biological processes were visualized using the ggplot2 package version 3.5.2 in R version 4.4.1 [34].

### 2.9. Statistical Analysis

Results are presented as mean ± standard error of the mean (SEM). All experiments included biological replicates (*n* ≥ 3) with technical replicates as specified in figure legends. Statistical significance was set at *p* < 0.05 for all analyses.

#### 2.9.1. Viral Replication Analysis

**Figure 1:**
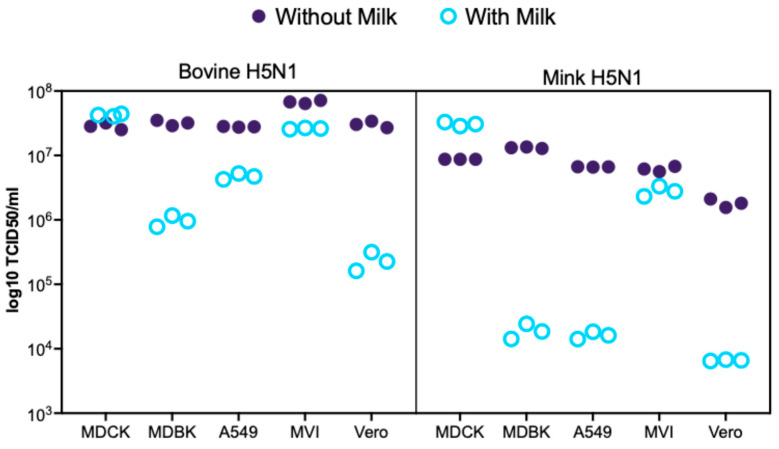
Replication of bovine- and mink-derived H5N1 viruses in mammalian cell lines with and without raw milk supplementation. Viral replication levels expressed as log_10_ TCID50 equivalents per mL from RT-qPCR targeting the influenza A matrix gene in MDCK, A549, MDBK, Vero, and MV1 cells at 24 h post-infection (MOI = 0.01) with bovine H5N1 (A/dairy cattle/Kansas/5/2024) or mink H5N1 (A/Mink/Spain/3691-8_22VIR10586-10/2022). Cells were incubated in standard medium (without milk) or raw milk–milk-supplemented medium (with milk). Data represent individual technical replicates (*n* = 3). Viral loads were calculated using a validated standard curve correlating Ct values to known viral titers (R^2^ = 0.9779, linear range 10^2^–10^8^ TCID50/mL). Results represent RNA levels as log_10_ TCID50 equivalents; correlation with infectious virus production requires validation.

Viral replication data (Figure 1, Supplementary Data) were analyzed using three-way ANOVA to assess the main effects and interactions of cell line (5 levels), H5N1 isolate (bovine vs. mink-derived), and milk supplement (present vs. absent) using GraphPad Prism version 10.6.0 (GraphPad Software, Inc., San Diego, CA, USA). The analysis evaluated seven F statistics: three main effects, three two-way interactions, and one three-way interaction. When significant main effects or interactions were detected (*p* < 0.05), post hoc multiple comparisons were performed using Tukey’s honestly significant difference (HSD) test to identify specific pairwise differences between treatment groups with family-wise error rate correction.

#### 2.9.2. RNA-Sequencing Differential Expression Analysis

Differential gene expression analysis was performed using DESeq2 (version 1.42.1) in R Studio (version 2025.05.1+513). Raw count data were filtered using independent filtering with default parameters to remove genes with low expression. Differential expression was determined using false discovery rate (FDR) < 0.05 and adjusted *p*-value < 0.05 using the Benjamini–Hochberg method for multiple testing correction. Gene set enrichment analysis (GSEA) was performed on significantly differentially expressed genes to identify enriched biological pathways.

## 3. Results

### 3.1. Replication Efficiency of HPAIV H5N1 Strains in Mammalian Cell Lines Is Affected by the Addition of Raw Milk

The replication efficiency of bovine-derived H5N1 and mink-derived H5N1 clade 2.3.4.4b viruses was evaluated across five mammalian cell lines: MDCK (canine), MDBK (bovine), A549 (human), Vero (monkey), and MV1 (mink). Viral replication was quantified by RT-qPCR analysis of the influenza A virus Matrix gene at 24 h post-infection, with results expressed as TCID_50_ equivalents per milliliter using the standard curve relationship described in Methods (Figure 1, Supplementary data). The 24 h post-infection time point was selected based on viral kinetics observed in MDCK cells, where replication peaks at 24 hpi without causing extensive cell death [27].

Across all mammalian cell lines, the bovine-derived H5N1 virus demonstrated robust replication, showing consistently more efficient replication than mink-derived H5N1 under standard infection conditions (without milk), particularly in Vero and MV1 cells.

The effect of raw milk supplementation on viral replication was examined across five mammalian cell lines using both bovine- and mink-derived H5N1 isolates (Figure 1, Supplementary Data). Raw milk treatment produced cell-line- and isolate-specific effects on H5N1 replication. MDCK cells demonstrated minimal changes in viral RNA levels with bovine-derived H5N1 regardless of milk supplementation but showed enhanced replication when infected with mink-derivedH5N1 in the presence of milk. In contrast, other cell lines demonstrated more pronounced responses to milk treatment, with A549, MDBK, and Vero cells showing greater reductions in viral replication when infected with mink-derived H5N1 compared to bovine-derived H5N1. MV1 cells exhibited intermediate responses to milk supplementation. The differential effects observed across cell lines and between H5N1 isolates indicate that raw milk supplementation does not uniformly enhance H5N1 replication in vitro, with the magnitude and direction of effects being dependent on both the cellular host environment and viral strain characteristics.

### 3.2. Viral Genomic Adaptation Evident from Amino Acid Mutations

**Figure 2:**
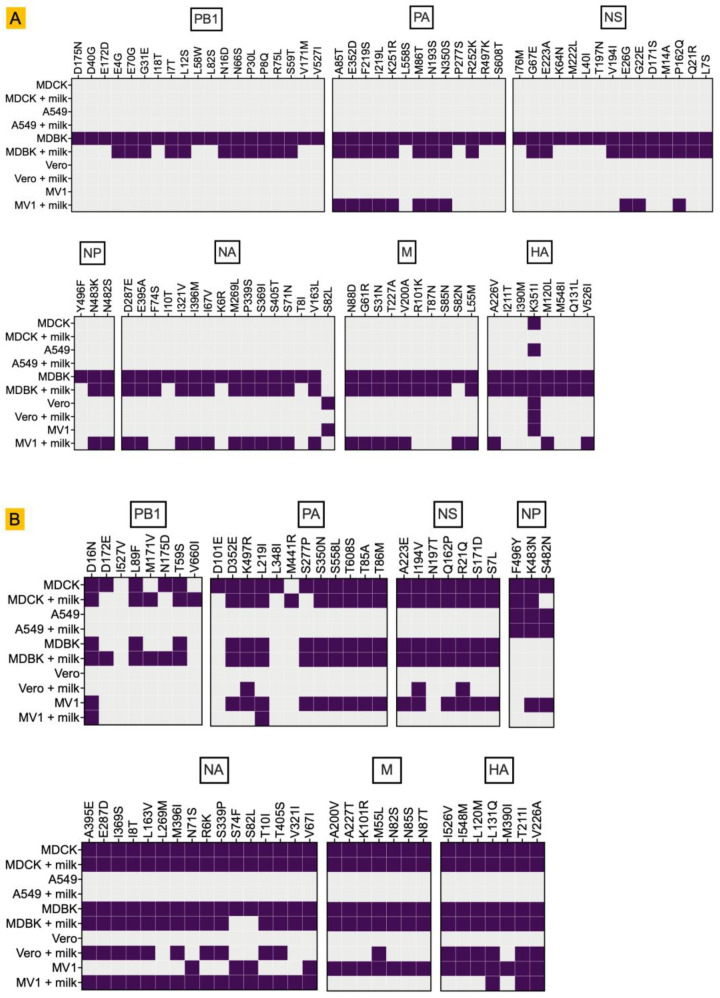
Host-and condition-specific amino acid substitutions in bovine- and mink-derived H5N1 viruses. Heatmaps of substitutions (≥5% frequency) in viral populations recovered 24 h post-infection from MDCK, A549, MDBK, Vero, and MV1 cells under standard (−milk) or raw milk-supplemented (+milk) conditions. (**A**) Bovine-H5N1 (A/dairy cattle/Kansas/5/2024). (**B**) Mink-H5N1 (A/Mink/Spain/3691-8_22VIR10586-10/2022). Purple boxes indicate the presence of the substitution.

Deep sequencing of RNA isolated from infected cells revealed distinct mutation profiles for both the bovine- and mink-derived H5N1 clade 2.3.4.4b isolates, with cell line–specific differences in their mutational landscapes. (Figure 2).

Amino acid substitutions of the bovine-derived H5N1 isolate were mainly detected in MDBK cells without milk (Figure 2A). MDBK cells supplemented with raw milk also had many but not all of these substitutions, indicating that components of raw milk modify the selective mutational pressures during replication. Interestingly, many of these mutations also emerged in MV1 cells supplemented with milk (specifically in PA, NA, and M), whereas none were detected in MV1 cells without milk. Notably, we consistently detected the HA K351I substitution across all cell lines, a change with no previous reports in the literature.

Mink-derived H5N1 showed a broad and uniform mutational profile across all host cells except A549 cells (Figure 2B). Most of the mutations in the PB1, PA, NS, NA, M, and HA segments of the isolate were detected in MDCK, MDBK, and MV1 cells, both with and without milk supplementation, as well as in Vero cells supplemented with milk. Notably, mutations in the NP segment were observed in A549, MDCK, and MV1 cells independent of milk supplementation, suggesting cell and strain-specific constraints on NP evolution. [35,36].

For both viruses, no mutations were detected in the PB2 segment across any of the cell lines.

### 3.3. Global Transcriptomic Changes Reveal Robust Antiviral Responses Dependent on Cell Type and Raw Milk

**Figure 3:**
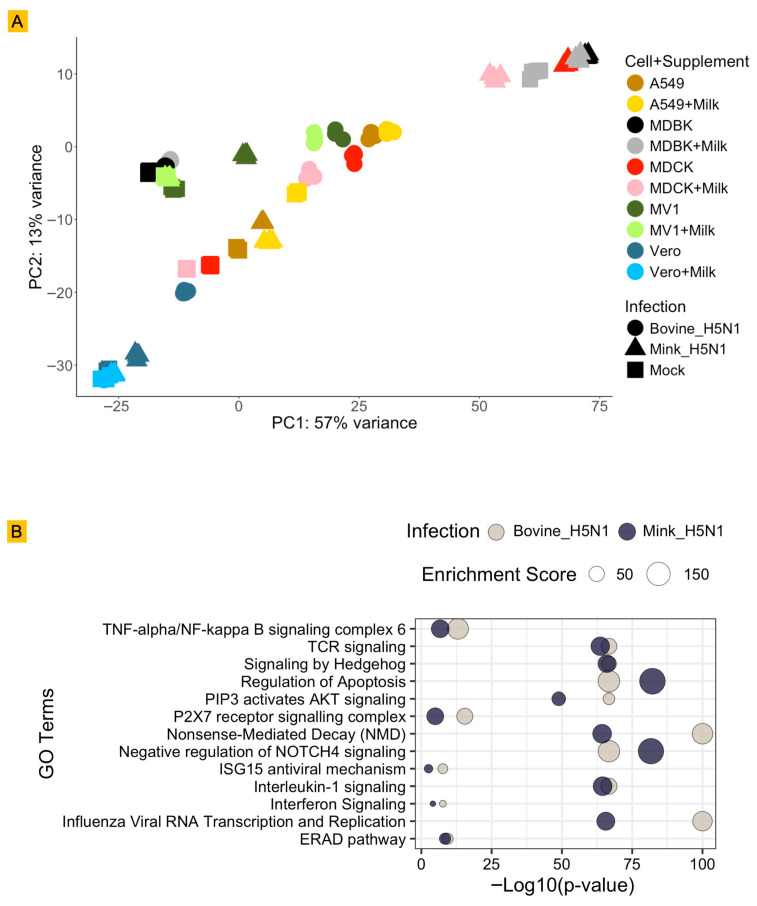
Transcriptional profiling of cell type- and environment-dependent responses to H5N1 infection. (**A**) Principal component analysis (PCA) of variance Stabilizing Transformation (VST)-normalized RNA-seq counts from A549, MDCK, MDBK, MV1, and Vero cells at 24 h post-infection with bovine-H5N1, mink-H5N1, or mock, with or without raw milk supplementation. Shapes denote infection type (bovine-H5N1 = circle; mink-H5N1 = triangle; mock = square) and colors indicate the cell lines with or without milk. (**B**) Gene Ontology (GO) enrichment for genes upregulated in bovine-H5N1 (gray) and mink-H5N1 (dark blue) infections. Points are sized by enrichment score and ordered by −log_10_ (*p*-value). Enrichment was computed in Metascape and visualized in R (ggplot2).

To comprehensively understand the host cellular response to H5N1 infection and the influence of raw milk, global transcriptomic changes were analyzed using RNA-Seq. A Principal Component Analysis (PCA) plot was generated to visualize the overall variance in gene expression profiles (Figure 3A).

The PCA plot clearly demonstrates that the primary driver of global gene expression changes is the infection status. PC1, which accounts for a substantial 57% of the total variance, distinctly separates mock-infected samples from H5N1-infected samples across all cell lines. While infection status was the dominant factor, PC2, explaining 13% of the variance, primarily differentiates samples based on cell line and the presence or absence of raw milk within the infected groups. This indicates that cell type and milk supplementation are secondary, yet significant, modulators of the host transcriptomic response. This suggests that while the core antiviral response is initiated by the viral infection, the specific cellular environment (i.e., the species origin of the cell line) and external factors, such as the supplementation of raw milk, alter the magnitude and nature of this response. This highlights the intricate complexity of host–virus–environment interactions at a molecular level.

The GO-term enrichment analysis of genes upregulated upon infection reveals both shared and strain-specific host responses (Figure 3B). Both H5N1 isolates activate canonical influenza-associated pathways, particularly those linked to viral RNA transcription/replication and endoplasmic reticulum–associated degradation (ERAD), reflecting a conserved transcriptional program supporting viral genome replication and protein quality control. However, the magnitude and statistical significance of these virus-specific pathways differed by strain (enrichment of processes related to viral transcription) and was comparatively greater in bovine-H5N1-infected cells, suggesting stronger engagement of host machinery required for viral replication and protein folding/degradation.

In addition, pathways governing innate immune and inflammatory signaling were more prominently enriched in the bovine-H5N1 response, indicating that this isolate elicits a heightened pro-inflammatory and interferon-driven transcriptional program in the cell systems analyzed here.

By contrast, mink-H5N1 infection was more strongly associated with enrichment of pathways regulating apoptosis as well as negative regulation of NOTCH4.

### 3.4. Modulation of Specific Host Gene Expression by H5N1 Infection and Raw Milk

To investigate whether milk modulates host genes implicated in influenza A virus infection and whether these effects differ between H5N1 strains, we curated a selected list of host genes from our RNA-seq dataset based on prior literature describing genes important for influenza A replication. This list was subsequently refined to include only genes with full annotation across all host species examined.

#### 3.4.1. Modulation of Genes Involved in Entry, Adsorption, and Uncoating

**Figure 4:**
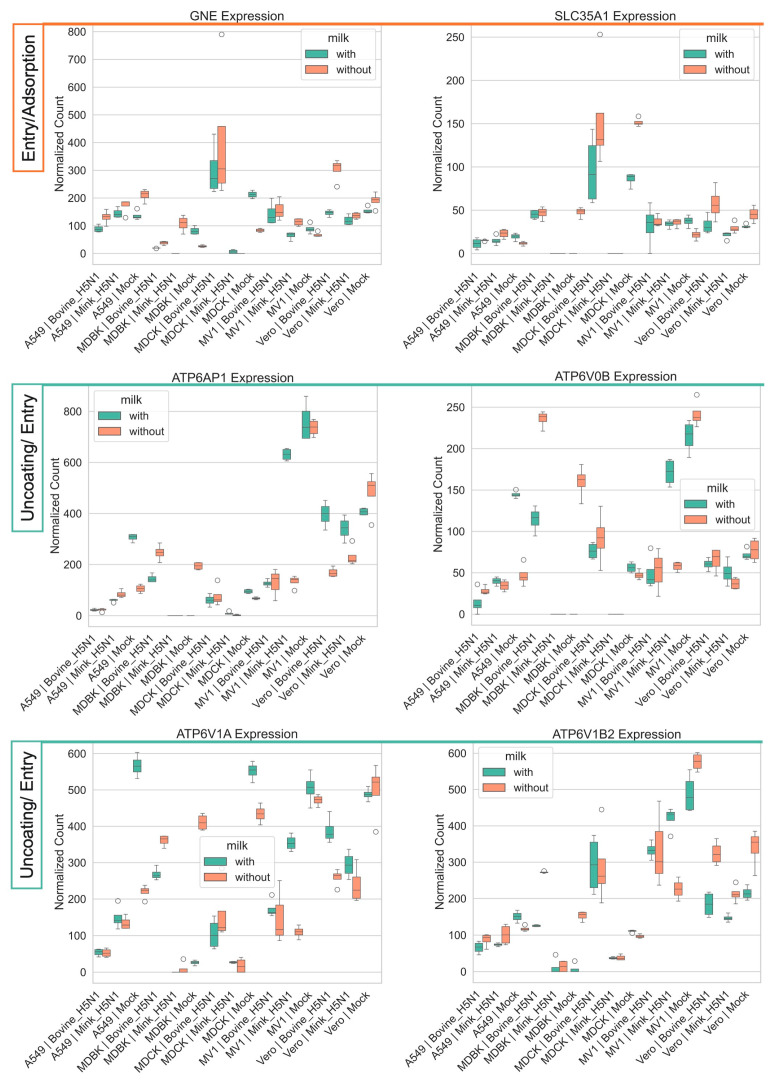
Impact of H5N1 strain and milk supplementation on host genes involved in influenza A entry, adsorption, and uncoating. VST normalized RNA-seq counts for six entry/adsorption/uncoating-associated genes (*GNE, SLC35A1, ATP1B1, ATP1B4, ATP1A1, ATP1B3*) were measured at 24 h post-infection in five mammalian cell lines (MDCK, MDBK, A549, MV1, and Vero). Cells were infected with either bovine-H5N1 or mink-H5N1 (MOI = 0.01) and incubated in infection medium with or without raw milk. Boxplots display the distribution of expression values (*n* = 3 technical replicates): box = interquartile range (25th–75th percentiles), horizontal line = median, whiskers = 1.5 × IQR, and individual points = values beyond whiskers.

Genes mediating early stages of viral entry and uncoating, including *GNE* and *SLC35A1* (involved in sialic acid biosynthesis and transport), along with vacuolar ATPase subunits *ATP6AP1*, *ATP6V1A*, *ATP6V0B*, and *ATP6V1B2* (essential for endosomal acidification and subsequent viral uncoating), are modulated by the presence of milk (Figure 4) [37,38,39,40,41]. However, the extent of these changes varied across cell types and viral strains as described in detail below.

Modulation of *GNE* and *SLC35A1* may impact sialylation patterns on the cell surface, thereby altering viral receptor availability [37,38]. In most cell lines, milk supplementation suppressed the expression of these genes, except in MDCK and MDBK mock controls. In MDCK cells infected with bovine-derived H5N1, both genes were markedly upregulated after infection and showed only a mild reduction in the presence of milk. A similar pattern of GNE expression was observed in MV1 cells, irrespective of infection with bovine- or mink-derived H5N1. The downregulation was more pronounced in cells infected with the bovine-derived strain, indicating greater sensitivity of the sialic acid biosynthetic pathway to this viral background during milk exposure.

Similarly, components of the vacuolar ATPase complex were broadly downregulated during milk exposure, except in MDCK and MDBK mock infection, with the strongest effect observed in bovine-H5N1-infected MDBK cells. Here, expression of *ATP6V0B*, *ATP6V1A*, and *ATP6V1B2* was substantially diminished in the presence of milk, potentially impairing endosomal acidification and viral uncoating efficiency [39,40,41].

#### 3.4.2. Modulation of Genes Involved in Genome Replication, Trafficking, and Protein Synthesis

**Figure 5:**
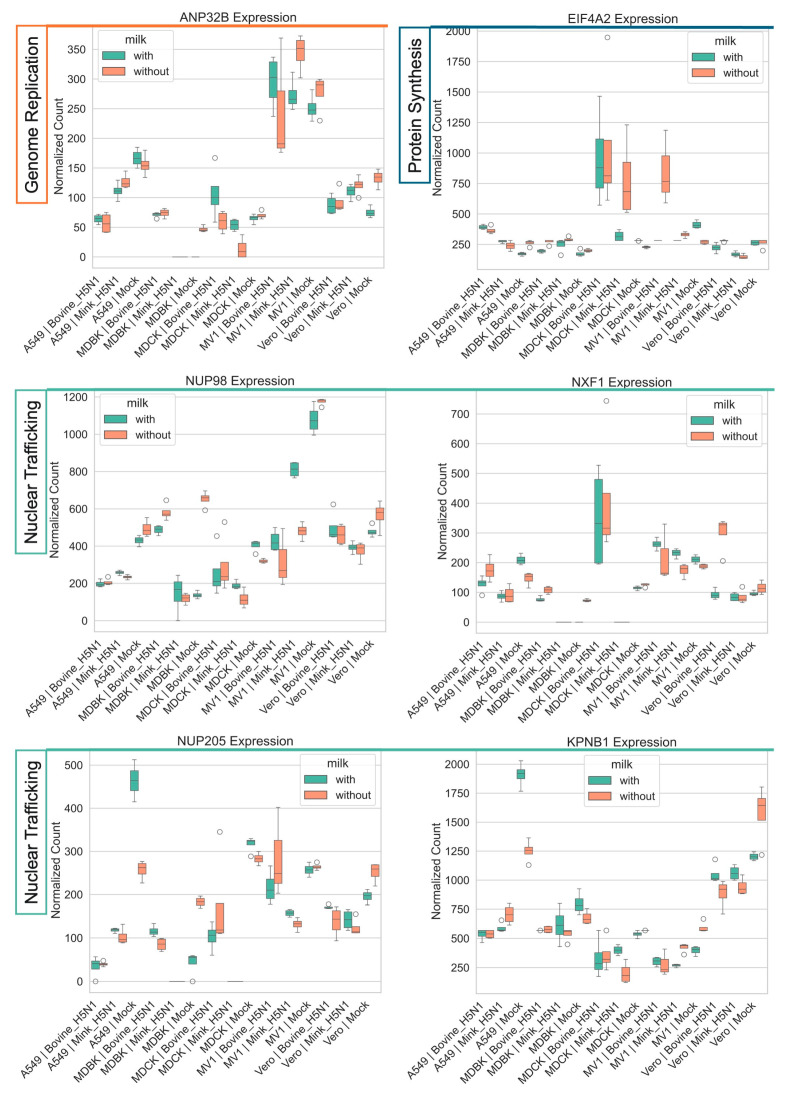
Impact of H5N1 strain and milk supplementation on host genes involved in influenza A genome replication, trafficking, and protein synthesis. VST normalized RNA-seq counts for genome replication, trafficking, and protein synthesis-associated genes (*ANP32B, EIF4A2, NUP98, NXF1, NUP205, KPNB1*) measured at 24 h post-infection in five mammalian cell lines (MDCK, MDBK, A549, MV1, and Vero). Cells were infected with either bovine-H5N1 or mink-H5N1 (MOI = 0.01) and incubated in infection medium with or without raw milk. Boxplots display the distribution of expression values (*n* = 3 technical replicates): box = interquartile range (25th–75th percentiles), horizontal line = median, whiskers = 1.5 × IQR, and individual points = values beyond whiskers.

Genes associated with influenza A viral genome replication, such as the human polymerase cofactor ANP32B, showed a complex and cell-type-specific response (Figure 5). In A549 cells, infection with both bovine- and mink-derived H5N1 induced marked downregulation of ANP32B. In contrast, infection with bovine-derived H5N1 upregulated ANP32B in MDCK and MDBK cells. In Vero cells, ANP32B expression was suppressed during infection, but this profile was reversed upon milk supplementation.

We also observed a generalized upregulation of genes involved in cellular protein synthesis and nuclear transport. Eukaryotic initiation factor 4A-2 (EIF4A2), a host protein essential for cap-dependent translation, was consistently upregulated upon infection across all cell lines except for Vero cells [42,43]. This upregulation was most pronounced in MDCK and MV1 cells, particularly with the more efficiently replicating bovine-H5N1 strain.

Several genes involved in nucleo-cytoplasmic trafficking, including NUP98, NUP205, and KPNB1, were broadly suppressed following H5N1 infection [44,45,46,47]. However, NUP98, NUP205, and NXF1 were upregulated in MDBK infected with bovine-H5N1. KPNB1 (Importin-β1), which mediates nuclear import of viral proteins, was downregulated in all infected cell lines regardless of viral strain [47]. In A549 cells, milk supplementation significantly upregulated NUP205 and KPNB1.

#### 3.4.3. Modulation of Host Antiviral Responses

**Figure 6:**
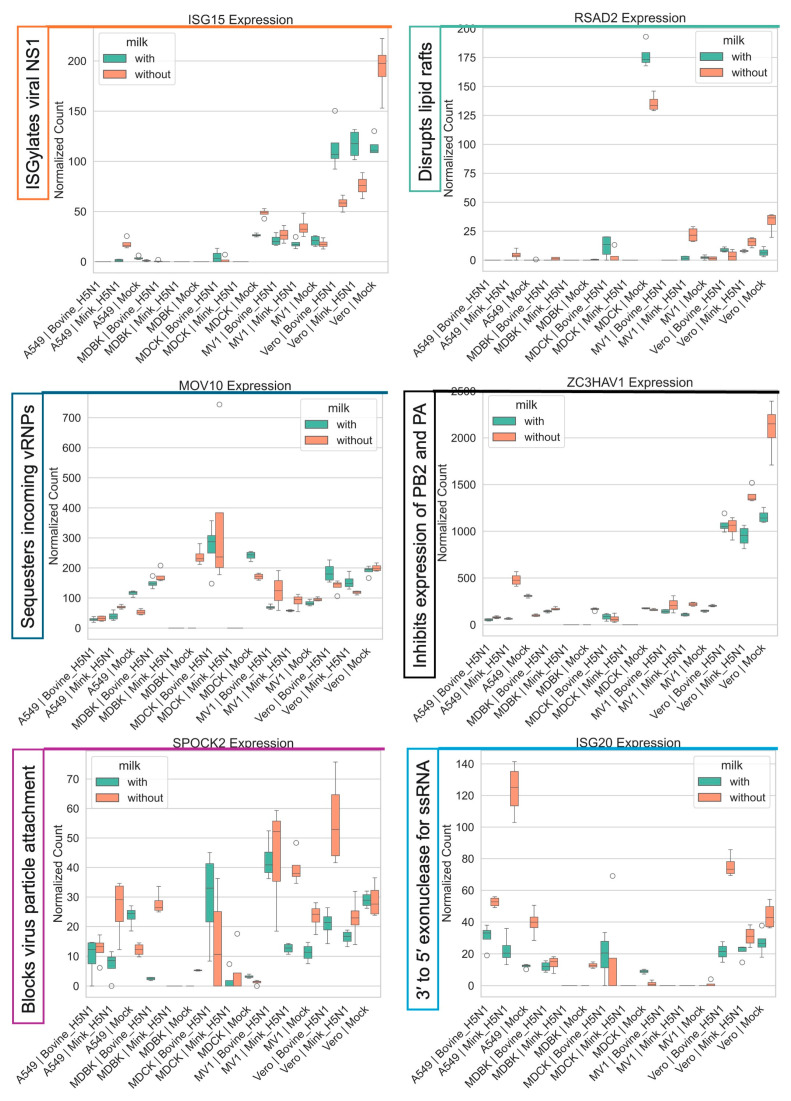
Impact of H5N1strain and milk supplementation on host genes involved in host antiviral responses against influenza A virus. VST normalized RNA-seq counts for host antiviral responses-associated genes (*ISG15, RSAD2, MOV10, ZC3HAV1, SPOCK2, ISG20*) measured at 24 h post-infection in five mammalian cell lines (MDCK, MDBK, A549, MV1, and Vero). Cells were infected with either bovine-H5N1 or mink-H5N1 (MOI = 0.01) and incubated in infection medium with or without raw milk. Boxplots display the distribution of expression values (*n* = 3 technical replicates): box = interquartile range (25th–75th percentiles), horizontal line = median, whiskers = 1.5 × IQR, and individual points = values beyond whiskers.

**Figure 7:**
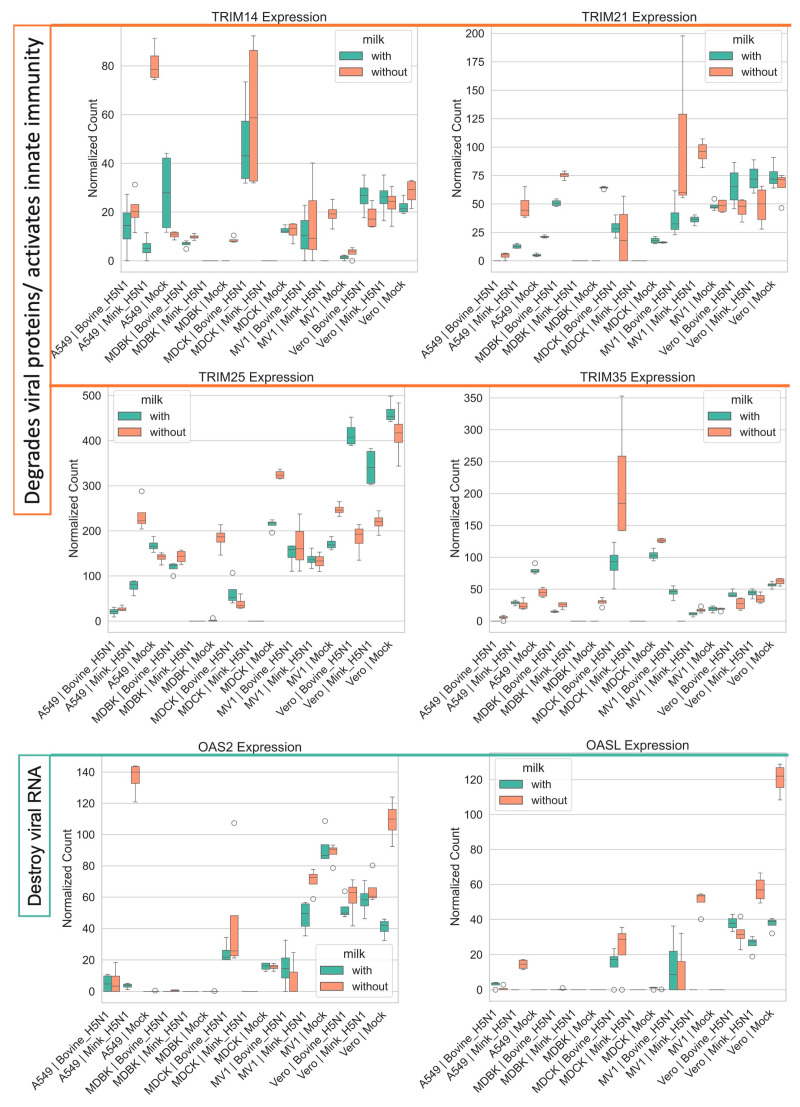
Impact of H5N1 strain and milk supplementation on host genes involved in host antiviral responses against influenza A virus. VST normalized RNA-seq counts for host antiviral responses-associated genes (*TRIM14, TRIM21, TRIM25, TRIM35, OAS2, OASL*) measured at 24 h post-infection in five mammalian cell lines (MDCK, MDBK, A549, MV1, and Vero). Cells were infected with either bovine-H5N1 or mink-H5N1 (MOI = 0.01) and incubated in infection medium with or without raw milk. Boxplots display the distribution of expression values (*n* = 3 technical replicates): box = interquartile range (25th–75th percentiles), horizontal line = median, whiskers = 1.5 × IQR, and individual points = values beyond whiskers.

Infection with both bovine-H5N1 and mink-H5N1 triggered a robust and widespread interferon-stimulated gene (ISG) response. However, the magnitude and specific targets of this response were modulated by cell type, viral strain, and the presence of raw milk (Figure 6 and Figure 7).

The key antiviral effector ISG15 was strongly downregulated in Vero cells; however, milk supplementation normalized its expression across all conditions (Figure 6). Similarly, the expression of RSAD2 (Viperin), an antiviral protein that inhibits viral budding and release, was decreased in Vero cells following infection, whereas milk supplementation restored its expression [48]. A similar pattern was observed for the MOV10 and ZC3HAV1 genes, host factors that restrict influenza replication; their induction was attenuated by milk in A549 cells [49]. In contrast, the expression of SPOCK2 and ISG20 was upregulated following infection with both bovine- and mink-derived H5N1.

The tripartite motif (TRIM) family of E3 ubiquitin ligases, which play a critical role in innate immunity by targeting viral proteins for degradation, also showed differential regulation (Figure 7) [50,51,52,53,54]. TRIM25, a key regulator of RIG-I signaling, was significantly downregulated in all infected cell lines, except in A549 cells infected with mink-derived H5N1 [53]. Interestingly, in Vero cells, milk supplementation reversed the infection-induced downregulation of TRIM25. In contrast, TRIM21, an antibody-targeting intracellular restriction factor, and TRIM14, a positive regulator of antiviral signaling, were broadly upregulated across cell lines, except in Vero cells, where both were downregulated, but milk supplementation restored their expression [50,51,54].

Finally, the expression of genes involved in viral RNA degradation, including OAS2 and OASL, varied substantially across cell lines and conditions (Figure 7) [55]. In A549 cells, infection with either bovine-or mink-derived H5N1 led to upregulation of these genes, with the strongest response observed in mink H5N1-infected cells without milk supplementation. In contrast, MDBK cells showed consistently low expression across all conditions. MDCK cells displayed moderate expression, with slightly higher levels during bovine-H5N1 infection. In MV1 and Vero cells, both genes were downregulated following infection.

#### 3.4.4. Host Signaling, Trafficking, and Assembly Pathways

We also investigated the transcriptomic changes in genes related to general host signaling pathways and intracellular trafficking, which are often co-opted or disrupted during viral replication.

**Figure 8:**
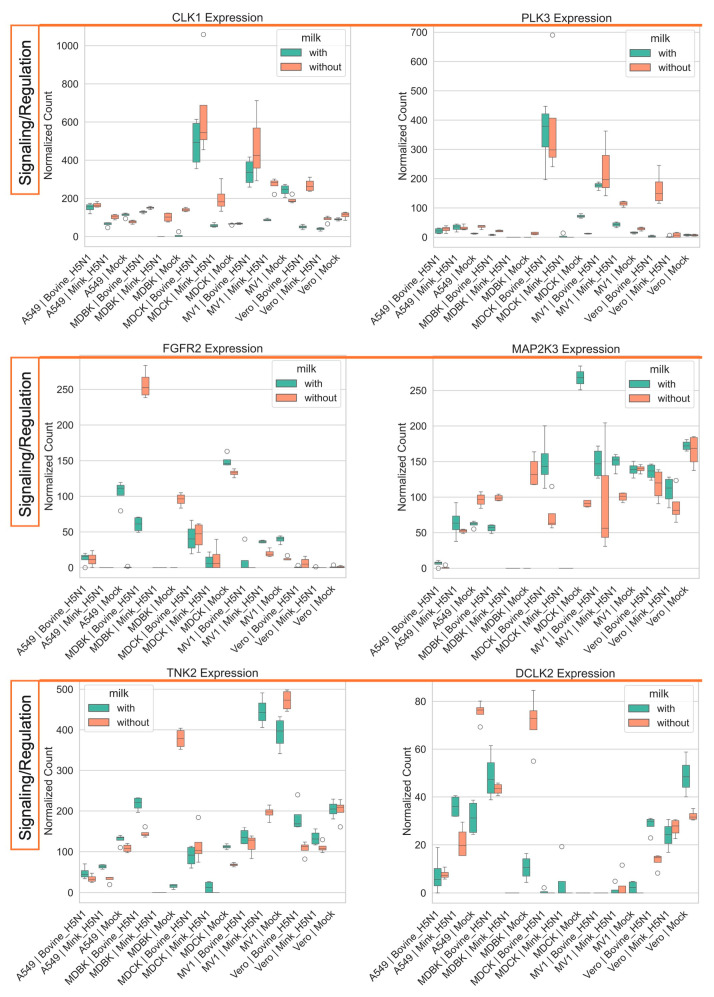
Impact of H5N1 strain and milk supplementation on host genes involved in host signaling. VST normalized RNA-seq counts for genes associated with host signaling (*CLK1, PLK3, FGFR2, MAP2K3, TNK2, DCLK2*) measured at 24 h post-infection in five mammalian cell lines (MDCK, MDBK, A549, MV1, and Vero). Cells were infected with either bovine-H5N1 or mink-H5N1 (MOI = 0.01) and incubated in infection medium with or without raw milk. Boxplots display the distribution of expression values (*n* = 3 technical replicates): box = interquartile range (25th–75th percentiles), horizontal line = median, whiskers = 1.5 × IQR, and individual points = values beyond whiskers.

Infection with H5N1 viruses induced differential regulation of signaling-related genes. CLK1 and PLK3, protein kinases involved in cell cycle regulation and signaling, were upregulated during bovine-derived H5N1 infection [56,57]. In contrast, FGFR2, MAP2K3, and TNK2, which are associated with growth factor and inflammatory signaling, were downregulated in a cell type-specific manner [58,59]. Milk supplementation upregulated these genes in mock-infected cells and produced only minor stimulating effects in infected cells (Figure 8).

**Figure 9:**
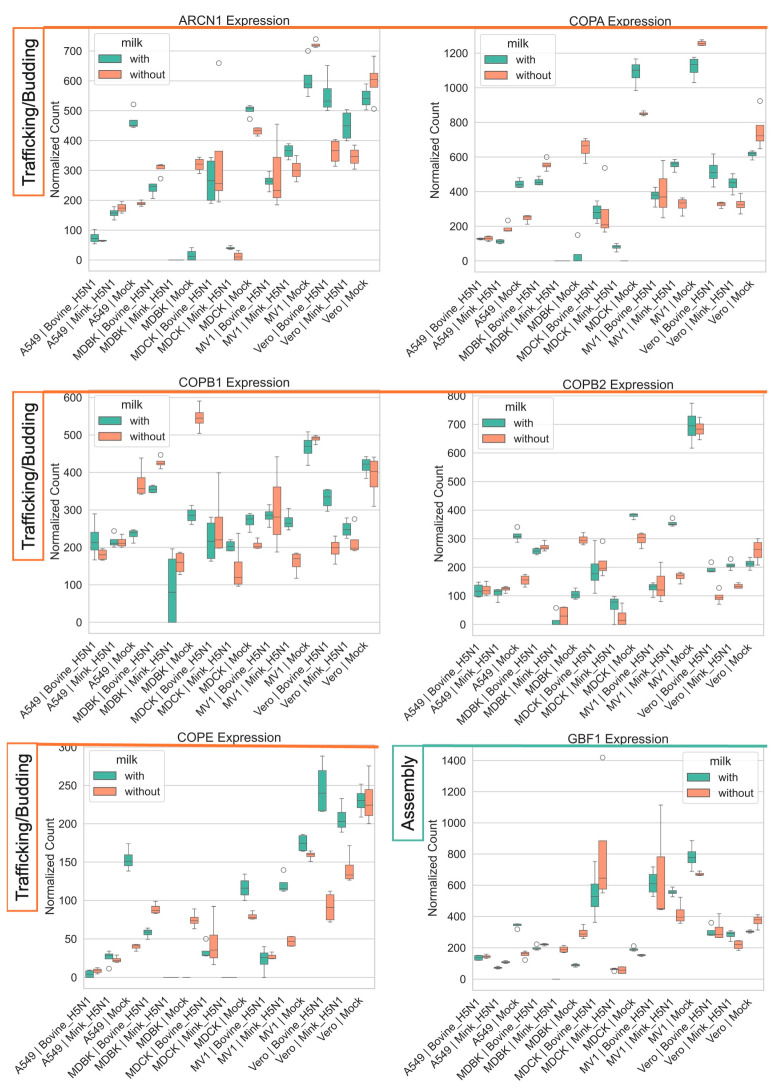
Impact of H5N1 strain and milk supplementation on host genes involved in virus assembly and trafficking. VST normalized RNA-seq counts for host genes associated with virus assembly and trafficking (*ARCN1, COPA, COPB1, COPB2, COPE, GBF1*) measured at 24 h post-infection in five mammalian cell lines (MDCK, MDBK, A549, MV1, and Vero). Cells were infected with either bovine-H5N1 or mink-H5N1 (MOI = 0.01) and incubated in infection medium with or without raw milk. Boxplots display the distribution of expression values (*n* = 3 technical replicates): box = interquartile range (25th–75th percentiles), horizontal line = median, whiskers = 1.5 × IQR, and individual points = values beyond whiskers.

Finally, H5N1 infection consistently downregulated genes central to the host intracellular trafficking machinery, particularly those involved in the COPI vesicle-mediated retrograde transport pathway, with more pronounced effects observed during mink-derived H5N1 infection compared to bovine-derived H5N1 (Figure 9) [41,60]. Similarly, the ARF GTPase-activating protein GBF1 was suppressed across all cell lines following infection. ARCN1, a gene involved in protein transport to the Golgi, was also downregulated alongside other COPI-related genes. Milk supplementation partially restored the expression of several COPI genes, mitigating the infection-induced suppression [61].

## 4. Discussion

The emergence of HPAI H5N1 in dairy cattle presents an unprecedented animal and public health challenge, driven by a novel transmission route involving infected raw milk [18,19,20]. Our study was designed to provide a detailed molecular understanding of how two distinct H5N1 strains, one from a recent bovine outbreak and one from a prior mink outbreak, interact with mammalian cells in the presence of raw milk [13,26]. The findings reveal a complex interplay between host cell type, viral strain, and the milk environment, highlighting a multifaceted interaction.

Our results confirm that the bovine-derived H5N1 strain generally replicates more efficiently across the mammalian cell line panel compared to the mink-derived strain, except in specific conditions such as mink-H5N1 infection with milk supplementation in MDCK cells. This enhanced replication of bovine-H5N1 is consistent with the broad dissemination and host range expansion of the bovine-H5N1 virus and underscores its inherent capacity for mammalian infection. The genetic data support this conclusion, with extensive viral genomic diversification observed across all eight viral segments during cell culture replication. Deep sequencing revealed distinct mutation profiles between the two viral strains, with bovine-derived H5N1 showing particularly high mutational diversity in MDBK cells, while mink-derived H5N1 displayed broader mutation patterns across multiple cell lines. Notably, we consistently detected the novel HA K351I substitution across all cell lines—a change with no previous reports in the literature. The observation that different cell lines and culture conditions (including milk supplementation) appear to influence the mutational landscape suggests that the cellular environment may theoretically modulate viral evolutionary pressures. Importantly, no mutations were detected in the PB2 segment across all conditions, indicating segment-specific constraints on viral evolution in these mammalian cell culture systems [62,63].

A notable observation from our in vitro studies is the apparent modulation of host antiviral gene expression by raw milk components. For example, milk supplementation was found to reduce the induction of key antiviral genes such as TRIM25 and RSAD2 in a cell-type-specific manner. TRIM25 is a critical sensor in the RIG-I pathway, and its suppression could hinder the initiation of a robust interferon response [52,53]. However, raw milk did not enhance viral replication in vitro; it rather exerted a consistent, albeit modest, inhibitory effect. The basis of this observation is revealed in our transcriptomic analysis, indicating that milk components can specifically modulate the host’s gene expression landscape, often in ways that could be detrimental to the virus. Notably, milk supplementation led to downregulation of host trafficking genes, suggesting disruption of essential cellular machinery. In addition, the milk-induced downregulation of sialic acid biosynthesis genes (GNE, SLC35A1) and endosomal acidification genes (ATP6V0B) could alter viral entry and uncoating processes. These findings demonstrate that milk supplementation produces complex, context-dependent effects on H5N1 replication, with enhancement observed in specific virus-cell combinations (notably mink-H5N1 in MDCK cells) while showing suppressive effects in others. This suggests that milk components may influence viral-host interactions through mechanisms that require further investigation using infectious virus assays and in vivo validation.

While these in vitro findings provide mechanistic insights, their translation to farm-level transmission requires careful interpretation. The modulation of ISG responses in cell culture may parallel processes in mammary epithelial cells during natural infection, where high viral loads (10^6^–10^8^ genome copies/mL) have been documented in milk from infected cattle. The context-dependent effects we observed, where milk enhanced viral RNA in specific combinations while suppressing it in others, may reflect the complex microenvironment within infected mammary glands. However, in vivo conditions involve additional factors absent from cell culture: circulating antibodies, immune cell infiltration, milk composition variability, and dynamic flow conditions during lactation. Therefore, controlled infection studies in lactating animals are essential to validate whether our observed in vitro effects translate to altered transmission dynamics in dairy environments.

In conclusion, this study provides a comprehensive molecular snapshot of H5N1 replication in mammalian cells with and without milk supplementation. We demonstrated that the bovine-derived H5N1 strain exhibits more robust replication across diverse mammalian cell lines than the mink-derived H5N1 and undergoes extensive genomic diversification, including the novel HA K351I substitution consistently detected across all cell lines. Most importantly, we have shown that raw milk, rather than acting as a simple enhancement or suppression factor, significantly modulates the host’s cellular response in complex, context-dependent ways. These effects vary by cell line and virus isolate combination, sometimes creating conditions that could theoretically favor viral replication (as observed with mink-H5N1 in MDCK cells), while in other contexts appearing to restrict viral activity through ISG suppression and trafficking disruption.

## 5. Future Research Directions

Based on these findings, we propose a systematic research roadmap to validate and extend these observations. Critical future studies include conducting viral kinetics studies and validation of virus progeny with observed mutations, alongside targeted fractionation studies to isolate specific milk components (lactoferrin, immunoglobulins A and G, oligosaccharides, fat globule membranes) and identify which factors drive ISG modulation in physiologically relevant primary mammary epithelial cells. Mechanistic investigations should elucidate molecular pathways by which identified milk components interact with innate immune signaling cascades (RIG-I/MDA5, TLR, cGAS-STING), employ reverse genetics to functionally validate the novel HA K351I mutation and other emerging variants, and assess whether milk-mediated ISG suppression translates to enhanced viral fitness across multiple replication cycles using longitudinal passage studies. Translational validation through bovine mammary organoid models, controlled infection studies in lactating mice or dairy cattle, and systematic analysis of milk samples from H5N1-infected cattle will be essential to determine correlations between viral load, milk composition profiles, and transmission efficiency under authentic field conditions. This hypothesis-generating study establishes that milk components may influence H5N1-host interactions through multiple mechanisms, providing testable hypotheses and mechanistic foundations for targeted investigations that could support farm and public health interventions to mitigate H5N1 transmission risks in dairy environments.

## 6. Limitations

Despite providing valuable insights, this study has several limitations that warrant consideration for future research.

First, our study measured viral RNA levels rather than infectious virus production—the ultimate determinant of transmission risk. RNA abundance may not correlate with infectivity, particularly when host antiviral responses are modulated by milk components. This distinction is crucial for assessing real-world transmission implications. Second, the study primarily focused on a single time point (24 h post-infection) for viral replication and transcriptomic analysis. While this captures acute responses, viral kinetics and host responses are dynamic processes that evolve over extended infection periods. Evaluating additional time points could provide a more comprehensive understanding of infection progression, long-term adaptive processes relevant to sustained farm infections, and the temporal influence of milk. Third, we used commercially processed raw milk, which may differ substantially from fresh milk produced by H5N1-infected cattle in composition, bioactivity, and immunomodulatory factor concentrations.

The use of immortalized mammalian cell lines, while controlled, may not fully recapitulate the complex physiological environment of a living organism or the specific cellular architecture and immune responses found in vivo. The findings from cell culture models may not directly translate to the full complexity of H5N1 infection and transmission in dairy cattle or other mammals, where circulating antibodies, immune cell infiltration, and tissue-specific immune responses play critical roles.

Raw milk is a highly complex biological fluid containing a vast array of proteins, fats, carbohydrates, immune cells, and microbial components. This study treated milk as a single supplement, and the specific components responsible for the observed modulatory effects on host gene expression remain unidentified. Dissecting the individual roles of various milk constituents would require further targeted experimentation.

While RNA-Seq provides a powerful global view of gene expression changes, it does not directly measure protein levels or their functional activity. Downregulation of a specific mRNA does not always correlate with a proportional decrease in its corresponding protein, nor does it guarantee altered protein levels. Several post-transcriptional mechanisms may maintain protein homeostasis despite mRNA changes, including (1) microRNA-mediated regulation that can alter translation efficiency independent of mRNA levels; (2) differential protein stability and degradation rates that vary substantially between proteins; (3) compensatory translation mechanisms that may upregulate protein synthesis when mRNA levels decrease; (4) ribosome occupancy and translation efficiency variations that are not reflected in total mRNA abundance; and (5) post-translational modifications that affect protein function without altering protein levels. These regulatory layers are particularly relevant for highly regulated genes encoding translation machinery components, nuclear transport proteins, metabolic enzymes, and signaling components. Therefore, our observed transcriptional changes should be interpreted as indicators of potential protein-level effects requiring validation rather than definitive evidence of altered protein abundance or function. Future studies incorporating ribosome profiling, polysome-associated mRNA analysis, targeted protein quantification (Western blot, proteomics), and functional assays would be essential to validate whether the transcriptional signatures we identified translate to biologically meaningful changes in protein levels in infected cells. 

The study’s conclusions, particularly regarding the impact of milk on viral adaptation and host immunity, would significantly benefit from validation in relevant animal models (e.g., lactating dairy cattle, susceptible small mammals). Such in vivo studies are crucial to confirm the physiological relevance of the in vitro observations.

While two distinct H5N1 strains (bovine- and mink-origin) were analyzed, the diversity of circulating H5N1 viruses is vast. Studying a broader panel of H5N1 isolates from various hosts and geographical regions could reveal more generalized or unique patterns of virus-host interaction and milk modulation.

The study observed rapid viral adaptation, but it did not employ reverse genetics or other molecular tools to directly link specific mutations to altered phenotypes (e.g., replication efficiency, milk sensitivity). Such experiments would provide additional evidence for the role of the observed mutations.

The limitations of this hypothesis-generating in vitro study underscore the need for multi-system validation before drawing definitive conclusions about H5N1 transmission in dairy environments. Nevertheless, our findings provide testable hypotheses and mechanistic foundations for targeted in vivo investigations that could inform evidence-based risk mitigation strategies.

## Figures and Tables

**Table 1:** Cell lines, animal species, reference genome assemblies, and accession numbers used for RNA-seq alignment.

Cell Line/Virus Isolate	Species	Reference Genome (Assembly and Version)	Source/Accession Number
MDCK	*Canis lupus familiaris*	CanFam3.1	NCBI RefSeq assembly accession GCF_000002285.3
MDBK	*Bos taurus*	ARS-UCD1.2	NCBI RefSeq assembly accession GCF_002263795.1
A549	*Homo sapiens*	GRCh38.p14	NCBI RefSeq assembly accession GCF_000001405.15
MV1	*Neogale vison* (*American mink*)	ASM_NN_V1 (Mustela_vison-1.0)	NCBI RefSeq assembly accession GCF_020171115.1
Vero	*Chlorocebus sabaeus* (*Green monkey*)	Chlorocebus_sabeus 1.1 (chlSab2)	NCBI RefSeq assembly accession GCF_000409795.2
Bovine H5N1 (A/dairy cattle/Kansas/5/2024)	*Influenza A virus*		NCBI GenBank ID PP732373-80
Mink H5N1 (A/Mink/Spain/3691-8_22VIR10586-10/2022)	*Influenza A virus*		GISAID ID EPI2220590-97

## Data Availability

Supplementary data is provided with the manuscript.

## Supplementary Materials

The following supporting information can be downloaded at: https://www.mdpi.com/article/10.3390/life15101625/s1, Supplementary Data: Strain- and Cell-Line Specific Responses to H5N1 Infection with or without Raw Milk Supplement.
